# Complete Genome Sequence of *Hyphomicrobium nitrativorans* Strain NL23, a Denitrifying Bacterium Isolated from Biofilm of a Methanol-Fed Denitrification System Treating Seawater at the Montreal Biodome

**DOI:** 10.1128/genomeA.01165-13

**Published:** 2014-01-16

**Authors:** Christine Martineau, Céline Villeneuve, Florian Mauffrey, Richard Villemur

**Affiliations:** INRS-Institut Armand-Frappier, Laval, Québec, Canada

## Abstract

*Hyphomicrobium nitrativorans* strain NL23 has been isolated from the biofilm of a denitrification system treating seawater. This strain has the capacity to denitrify using methanol as a carbon source. Here, we report the complete genome sequence of this strain in an effort to increase understanding of the function of this bacterium within the biofilm.

## GENOME ANNOUNCEMENT

Members of the genus *Hyphomicrobium* (class *Alphaproteobacteria*) are restricted facultative methylotrophs that reproduce by budding at the tip of a polar prostheca (1). They are ubiquitous in water and soil but can also be found in sewage treatment plants. Some strains are characterized by their denitrification capacities (2–5). Genome sequences are publicly available for *Hyphomicrobium denitrificans* ATCC 51888 (6), *Hyphomicrobium denitrificans* 1NES1 (7), *Hyphomicrobium* sp. strain MC1 (8), and *Hyphomicrobium zavarzinii* ATCC 27496 (GenBank accession number ARTG00000000). In previous work, we isolated strain NL23 from the denitrifying biofilm of a methanol-fed denitrification system treating the seawater of the St. Lawrence mesocosm at the Montreal Biodome, Canada (9). The strain was recently described as a new species, and the name *Hyphomicrobium nitrativorans* has been validated (10). Here, we report the complete genome sequence of this strain, which we determined in an effort to understand the function of this bacterium within the denitrifying biofilm from which it was isolated.

Sequencing of the genome was performed using the Roche Genome Sequencer FLX system and titanium chemistry (paired ends with an insert size of 8 kb). Primary assembly of the sequencing reads was carried out with Newbler gsAssembler v 2.5.3 (Roche) to generate scaffolds from the 454 paired-end library. A total of 354,899 reads were obtained (104,886 with paired-end reads), for 22× coverage of the genome. After multiple rounds of gap-closing steps using CONSED version 20.0 (11), a single contig was obtained. The genome sequencing and assembly were performed at the Plateforme d’Analyses Génomiques of the Institut de Biologie Intégrative et des Systèmes (IBIS-Université Laval, Québec, Canada). Nucleotide sequences of 19 remaining gaps (17 to 365 nt) in the genome sequence were determined by our group using PCR and Sanger sequencing of both strands. Genome annotation was added by the NCBI Prokaryotic Genome Annotation Pipeline (release 2013).

The complete genome sequence of *Hyphomicrobium nitrativorans* NL23 is composed of a single chromosome of 3,653,837 bp, with a GC content of 63.8% and 3,469 putative genes. There are 3 rRNA operons (5S, 16S, and 23S), 49 tRNAs, and 1 noncoding RNA. Crucial genes linked to denitrification, methylotrophy, and assimilation of formaldehyde by the serine pathway were identified (10).

The genome of *H. nitrativorans* NL23 was compared to the genome of its closest relative, *H. zavarzinii* ATCC 27496. The later has a genome with 4,651,795 bp and 4,350 genes. Sequence-based comparison of the two genomes in RAST (Rapid Annotation using Subsystem Technology) (12) identified 2,687 orthologous genes with an overall identity of 73.85% (amino acid sequence). An average nucleotide identity (ANI) of 82.48% was also calculated between the two genomes (http://enve-omics.ce.gatech.edu/ani/index) (13). Together with phenotypic characteristics and results from DNA-DNA hybridization (10), genomic comparison further confirms that the two strains belong to distinct species.

### Nucleotide sequence accession number.

The genome sequences and annotations of *Hyphomicrobium nitrativorans* strain NL23 have been deposited in GenBank under accession number CP006912.

## References

[1] Gliesche CG Fesefeldt A Hirsch P 2005 Genus *Hyphomicrobium* Stutzer and Hartleb 1898, 76^AL^, p 476–494 *In* Staley JT Bryant MP Pfennig N Holt JG (ed), Bergey's manual of systematic bacteriology. Williams and Wilkins, Baltimore, MD.

[2] Fesefeldt A Kloos K Bothe H Lemmer H Gliesche CG 1998 Distribution of denitrification and nitrogen fixation genes in *Hyphomicrobium* spp. and other budding bacteria. Can. J. Microbiol. 44:181–186. 10.1139/w97-139

[3] Kloos K Fesefeldt A Gliesche CG Bothe H 1995 DNA-probing indicates the occurrence of denitrification and nitrogen fixation genes in *Hyphomicrobium*. Distribution of denitrifying and nitrogen fixing isolates of *Hyphomicrobium* in a sewage treatment plant. FEMS Microbiol. Ecol. 18:205–213. 10.1111/j.1574-6941.1995.tb00177.x

[4] Urakami T Sasaki J Suzuki K-I Komagata K 1995 Characterization and description of *Hyphomicrobium denitrificans* sp. nov. Int. J. Syst. Bacteriol. 45:528–532. 10.1099/00207713-45-3-528

[5] Timmermans P Van Haute A 1983 Denitrification with methanol: fundamental study of the growth and denitrification capacity of *Hyphomicrobium* sp. Water Res. 17:1249–1255. 10.1016/0043-1354(83)90249-X

[6] Brown PJ Kysela DT Buechlein A Hemmerich C Brun YV 2011 Genome sequences of eight morphologically diverse *Alphaproteobacteria*. J. Bacteriol. 193:4567–4568. 10.1128/JB.05453-11 21705585 PMC3165538

[7] Venkatramanan R Prakash O Woyke T Chain P Goodwin LA Watson D Brooks S Kostka JE Green SJ 2013 Genome sequences for three denitrifying bacterial strains isolated from a uranium- and nitrate-contaminated subsurface environment. Genome Announc. 1(4):e00449-13. 10.1128/genomeA.00449-13 23833140 PMC3703601

[8] Vuilleumier S Nadalig T Ul Haque MF Magdelenat G Lajus A Roselli S Muller EE Gruffaz C Barbe V Médigue C Bringel F 2011 Complete genome sequence of the chloromethane-degrading *Hyphomicrobium* sp. strain MC1. J. Bacteriol. 193:5035–5036. 10.1128/JB.05627-11 21868803 PMC3165642

[9] Labbé N Juteau P Parent S Villemur R 2003 Bacterial diversity in a marine methanol-fed denitrification reactor at the Montreal Biodome, Canada. Microb. Ecol. 46:12–21. 10.1007/s00248-002-1056-6 12739074

[10] Martineau C Villeneuve C Mauffrey F Villemur R 2013 *Hyphomicrobium nitrativorans* sp. nov., isolated from the biofilm of a methanol-fed denitrification system treating seawater at the Montreal Biodome. Int. J. Syst. Evol. Microbiol. 63:3777–3781. 10.1099/ijs.0.048124-0 23667138

[11] Gordon D Abajian C Green P 1998 Consed: a graphical tool for sequence finishing. Genome Res. 8:195–202. 10.1101/gr.8.3.195 9521923

[12] Aziz RK Bartels D Best AA DeJongh M Disz T Edwards RA Formsma K Gerdes S Glass EM Kubal M Meyer F Olsen GJ Olson R Osterman AL Overbeek RA McNeil LK Paarmann D Paczian T Parrello B Pusch GD Reich C Stevens R Vassieva O Vonstein V Wilke A Zagnitko O 2008 The RAST server: Rapid Annotations using Subsystems Technology. BMC Genomics 9:75. 10.1186/1471-2164-9-75 18261238 PMC2265698

[13] Goris J Konstantinidis KT Klappenbach JA Coenye T Vandamme P Tiedje JM 2007 DNA–DNA hybridization values and their relationship to whole-genome sequence similarities. Int. J. Syst. Evol. Microbiol. 57:81–91. 10.1099/ijs.0.64483-0 17220447

